# Force encoding in muscle spindles during stretch of passive muscle

**DOI:** 10.1371/journal.pcbi.1005767

**Published:** 2017-09-25

**Authors:** Kyle P. Blum, Boris Lamotte D’Incamps, Daniel Zytnicki, Lena H. Ting

**Affiliations:** 1 Wallace H. Coulter Department of Biomedical Engineering, Emory University and Georgia Institute of Technology, Atlanta, Georgia, United States of America; 2 Center for Neurophysics, Physiology and Pathophysiology, Université Paris Descartes, Paris, France; 3 Department of Rehabilitation Medicine, Division of Physical Therapy, Emory University, Atlanta, Georgia, United States of America; Northeastern University, UNITED STATES

## Abstract

Muscle spindle proprioceptive receptors play a primary role in encoding the effects of external mechanical perturbations to the body. During externally-imposed stretches of passive, i.e. electrically-quiescent, muscles, the instantaneous firing rates (IFRs) of muscle spindles are associated with characteristics of stretch such as length and velocity. However, even in passive muscle, there are history-dependent transients of muscle spindle firing that are not uniquely related to muscle length and velocity, nor reproduced by current muscle spindle models. These include acceleration-dependent initial bursts, increased dynamic response to stretch velocity if a muscle has been isometric, and rate relaxation, i.e., a decrease in tonic IFR when a muscle is held at a constant length after being stretched. We collected muscle spindle spike trains across a variety of muscle stretch kinematic conditions, including systematic changes in peak length, velocity, and acceleration. We demonstrate that muscle spindle primary afferents in passive muscle fire in direct relationship to muscle *force-related* variables, rather than *length-related* variables. Linear combinations of whole muscle-tendon force and the first time derivative of force (dF/dt) predict the entire time course of transient IFRs in muscle spindle Ia afferents during stretch (i.e., lengthening) of passive muscle, including the initial burst, the dynamic response to lengthening, and rate relaxation following lengthening. Similar to acceleration scaling found previously in postural responses to perturbations, initial burst amplitude scaled equally well to initial stretch acceleration or dF/dt, though later transients were only described by dF/dt. The transient increase in dF/dt at the onset of lengthening reflects muscle short-range stiffness due to cross-bridge dynamics. Our work demonstrates a critical role of muscle cross-bridge dynamics in history-dependent muscle spindle IFRs in passive muscle lengthening conditions relevant to the detection and sensorimotor response to mechanical perturbations to the body, and to previously-described history-dependence in perception of limb position.

## Author summary

Proprioceptive sensory information is essential to movement, particularly in sensorimotor responses to external perturbations to the body–such as a push or bump–whether maintaining the posture of a limb, or during standing balance control. Here we show that rapid increase in resistive force of a passive muscle when stretched may cause enhanced sensory signals that facilitate the detection and response to sudden mechanical perturbations to the body. Our work is significant because these transient increases in muscle spindle firing have not been explained previously in terms of the classical explanation of muscle spindles encoding changes in muscle length and velocity. Our work suggests that a sense of muscle force may serve as a good proxy for muscle length in many conditions, but also increases sensory encoding of perturbations when our bodies are at rest. Further work may incorporate our findings to develop more accurate models of proprioceptive encoding that can better predict our sensory, motor, and perceptual responses to perturbation and how they are affected by neurological disorders that affect sensing and moving.

## Introduction

Proprioceptive sensory information is essential to movement, particularly in sensorimotor responses to external perturbations to the body–such as a push or bump–whether maintaining the posture of a limb, or during standing balance control [1]. Following a postural perturbation to standing balance, a transient pattern of corrective muscle activity follows the time course of the displacement, velocity, and acceleration of the body caused by the perturbation [2–5] that is impaired after proprioceptive loss [6, 7]. Thus, the transformation between mechanical events in the body due to an external perturbation and the transient firing of proprioceptive afferents is critical to understanding sensorimotor control of posture and balance [8, 9]. Muscle spindle proprioceptive receptors likely play a primary role in encoding the effects of perturbation on the body as they fire trains of action potentials during muscle stretch that vary as a function of experimentally-imposed muscle length and velocity [10–14]. However, our current understanding and computational models of muscle spindle sensory encoding are insufficient to account for several transient and history-dependent properties of muscle spindle firing rates observed experimentally, and that also appear to be important in postural control.

Muscle spindle instantaneous firing rates (IFRs) in response to muscle stretch are not uniquely related to muscle length and velocity, but exhibit transient, history-dependent features in conditions relevant to detection and sensorimotor responses to postural perturbations. If a muscle is held at a constant length for a period of time before being stretched, two history-dependent features in muscle spindle IFRs appear that are absent if the muscle has been moving directly prior to stretch: 1) an initial burst of action potentials at the onset of stretch, and 2) an elevated firing rate during constant velocity ramps [15]. Because muscles are maintained at a constant length during control of posture and balance, these history-dependent features likely play a critical function in the detection of and sensorimotor response to perturbations. Moreover, both the initial burst of muscle spindle sensory signals and the initial burst of muscle activity evoked by postural perturbations have been shown to vary with perturbation acceleration [2, 6, 16, 17]. A third history-dependent feature called rate relaxation, or rate adaptation, is observed where the tonic firing rate of the muscle spindle decreases when the muscle is held at a constant length after being stretched [18].

Current muscle spindle computational models fail to predict some history-dependent characteristics of muscle spindle IFRs [19–21]. The most commonly-used computational muscle spindle models use a muscle model to estimate stress in the intrafusal muscle fibers, found within muscle spindle sensory organs, based on measured changes in muscle length. The strain experienced by the sensory region of the fiber is then estimated with a linear model to predict responses to ramp-and-hold stretch perturbations [19–21]. Such models are generally capable of reproducing firing patterns in response to ramp stretches either when the muscle has been at rest, or when it has been moving prior to stretch, but they cannot account for spindle firing rates during transitions in movement state, which occur frequently in mammalian behavior [22]. For example, some muscle spindle models fail to capture the history-dependent nature of the initial burst based on the muscle state history [15, 19, 20], while others lack an initial burst altogether [21].

It has been proposed that the transient, history-dependent properties of muscle spindle firing are due to history-dependent muscle forces arising from non-steady-state cross-bridge dynamics [23–29]. Thus, the lack of history-dependence in muscle spindle models may be due to the use of phenomenological muscle force models (e.g., Hill-type muscle models) that do not account for history-dependence [19–21]. Experiments in muscle physiology have demonstrated history-dependence in muscle fiber forces, including a transient increase in force and the first time derivative of force (dF/dt) when an isolated, permeabilized muscle fiber activated in a calcium solution is stretched after being held isometrically. In intact muscles, a similar history-dependent transient force is observed when a muscle is stretched after being held isometrically, often referred to as short-range stiffness [30, 31]. This history-dependence is thought to arise from movement-dependent cycling of muscle cross-bridges [27–29, 32], and can be reproduced using models of cross-bridge kinetics [27–29]. Such muscle fiber force history-dependence has been implicated as a cause of history-dependent muscle spindle IFRs, but not yet demonstrated directly [15, 22, 23, 32]. Classic studies of muscle spindles also remarked on qualitative parallels between muscle spindle firing rates and muscle force transients during stretch [13, 23]. Moreover, transient features similar to force and dF/dt are evident in figures of rare recordings of receptor potentials in the encoding regions of muscles spindle afferents [33, 34]. However, technical limitations of the day prevented direct and quantitative testing of the hypothesis that transients and history-dependence in muscle spindle IFR encode muscle fiber force, beyond highlighting qualitative similarities between the traces.

Thus, towards improved computational models of proprioceptive encoding relevant to postural control, we performed quantitative analyses of muscle spindle spike trains to identify transformations between muscle mechanical events that best predict transient and history-dependent features of muscle spindle firing rates during muscle stretch. We directly tested the hypothesis that transient, history-dependent muscle spindle IFRs during stretch of a passive muscle encode information about muscle force. Here, we use “passive” to mean that the muscle is electrically-quiescent and lacks neural drive, as our experiments were conducted in deeply-anesthetized animals, but it is likely that muscle cross-bridge cycling is not completely absent. We collected muscle spindle firing rates across a wide variety of muscle stretch kinematic conditions in which a large range of initial burst amplitude, dynamic response, and rate relaxation characteristics were elicited. We tested whether pseudolinear combinations of muscle force-related versus length-related variables could better predict the temporal features of muscle spindle afferent IFRs including history-dependent firing characteristics. For each afferent recorded, we identified a unique set of parameters that best fit muscle state variables to spindle IFRs across all stretch conditions. Based on the goodness of fit and number of parameters, a model consisting of pseudolinear combinations of recorded muscle force and the first time derivative of force, dF/dt, was found to be the most likely model of muscle spindle IFRs across a wide range of passive muscle stretch conditions. To our knowledge, this is the first quantitative evidence that muscle spindle firing rates in transient, history-dependent conditions can be uniquely determined by muscle force. Our findings suggest that transient sensory information encoded by muscle spindle primary afferents in passive muscles is driven by transient mechanical properties of muscle cross bridges, as evidenced by history dependence in force- but not length-related variables.

## Results

### Response of muscle spindles Ia afferents to muscle stretch

All muscle afferents were confirmed to be muscle spindle primary afferents using classical criteria, i.e. axonal conduction velocity >74 m/s, resting discharge, and dynamic response to muscle stretch [10]. In five deeply anesthetized adult cats (2.5–3.5 kg), the distal extremity of the triceps surae muscles was isolated, mounted on a length servomotor, and force was monitored (see Materials and methods for further details). Stable recordings across a full protocol of diverse stretch perturbations were achieved in 12 afferents using sharp glass microelectrodes. Peak length, velocity, and acceleration were varied independently to elicit a variety of muscle force and muscle spindle firing responses.

**Fig 1:**
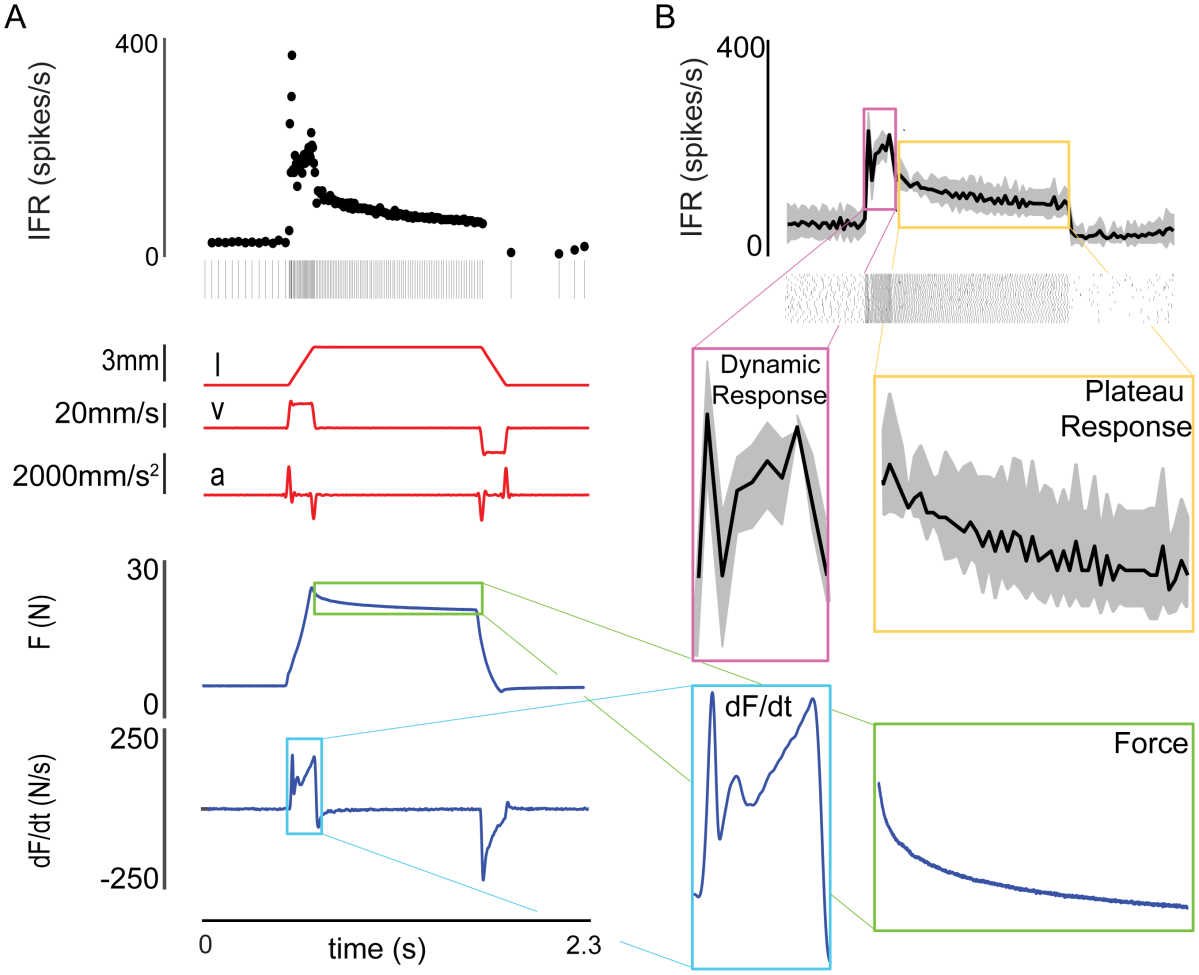
Similarity of muscle spindle IFRs and musculotendon force-related variables in during stretch. (A) An example ramp-hold-release length profile applied to the triceps surae at the calcaneus, and response of the musculotendon force and muscle spindle primary afferent spiking. The spike train from a single trial and corresponding instantaneous firing rate (IFR) are shown in black at the top as an example of a typical response. Perturbation kinematics (i.e. muscle length, velocity, and acceleration) are shown in red. The musculotendon response to this stretch (i.e. force and dF/dt) is shown in blue. (B) The similarities between muscle spindle IFR shown in black at the top (ensemble average of 20 trials with bin size of 20 ms) and the muscle force-related variables shown in A. Specifically, note the similarities between force (green box) and the plateau response of the muscle spindle (yellow box), and between rate change in force (light blue box) and the dynamic response of the muscle spindle afferent (magenta box). The examples shown are from afferent 2.

Muscle spindle IFRs showed striking similarities to musculotendon force-related variables, i.e., force and the first time derivative of force (dF/dt), over the entire time course of each stretch perturbation (Fig 1). IFRs exhibited history-dependent features, i.e., initial bursts and dynamic responses during ramp stretches, and rate relaxation during hold periods. These features were associated with changes in length- and force-related variables (Fig 1A red and blue traces, respectively), though the transient behaviors more closely resembled recorded force-related variables. During stretch, the initial burst and dynamic response closely resembled dF/dt. During post-lengthening hold, rate relaxation closely resembled musculotendon force (Fig 1B).

### Relationship of muscle spindle Ia initial burst magnitude to force- and length-related transients

To test potential variables encoded by the initial burst of spikes at stretch onset, we regressed maximum initial burst amplitude on corresponding peaks in force- and length-related transients, i.e. whole-muscle dF/dt and acceleration. For 6 afferents exhibiting consistent initial bursts (afferents 1, 2, 3, 5, 11, and 12), we performed a linear regression between the preceding peak in dF/dt or acceleration and peak initial burst amplitude across a minimum of 12 stretch trials in which muscle acceleration was varied systematically.

**Fig 2:**
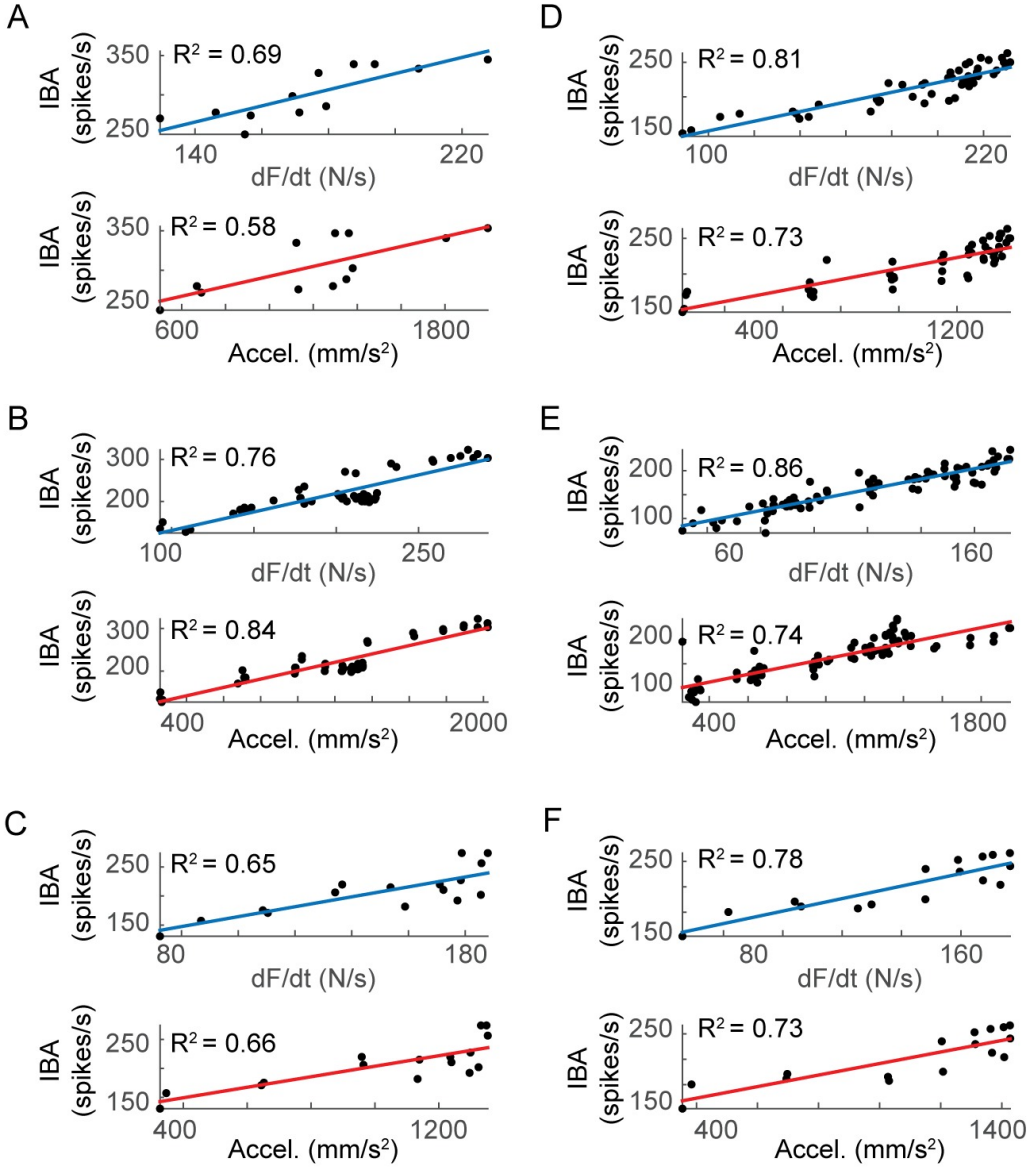
Linear regression of perturbation acceleration and muscle dF/dt to muscle spindle initial burst amplitude in 6 afferents. (A-F) Black dots represent the initial burst amplitude (IBA) versus either dF/dt or acceleration. Blue traces indicate linear regression of IBA on corresponding peaks in dF/dt. Red traces indicate linear regression of IBA on corresponding peaks in acceleration for the same trials. For all 12 cases (6 afferents, 2 regressions each), p < 0.05.

In these afferents, the peak initial burst amplitude (IBA: peak IFR during the initial burst) increased linearly with either dF/dt or acceleration (Fig 2). Either peak dF/dt or acceleration predicted similar amounts of variance in the initial burst amplitudes across all 6 afferents (R^2^ = 0.76 ± 0.08 with dF/dt; R^2^ = 0.71 ± 0.09 with acceleration). Peak initial burst firing rate increased with either dF/dt or acceleration in each of the 6 afferents (p < 0.05). Although peak acceleration amplitude predicted peak firing rate of the initial burst to a similar degree as peak dF/dt amplitude, acceleration exhibited a second peak during the return of the musculotendon to its initial length (e.g. Fig 1A) that did not have a corresponding peak in muscle spindle IFR nor whole muscle dF/dt.

### Relationship of muscle spindle Ia IFRs to musculotendon length- and force-related variables

**Fig 3:**
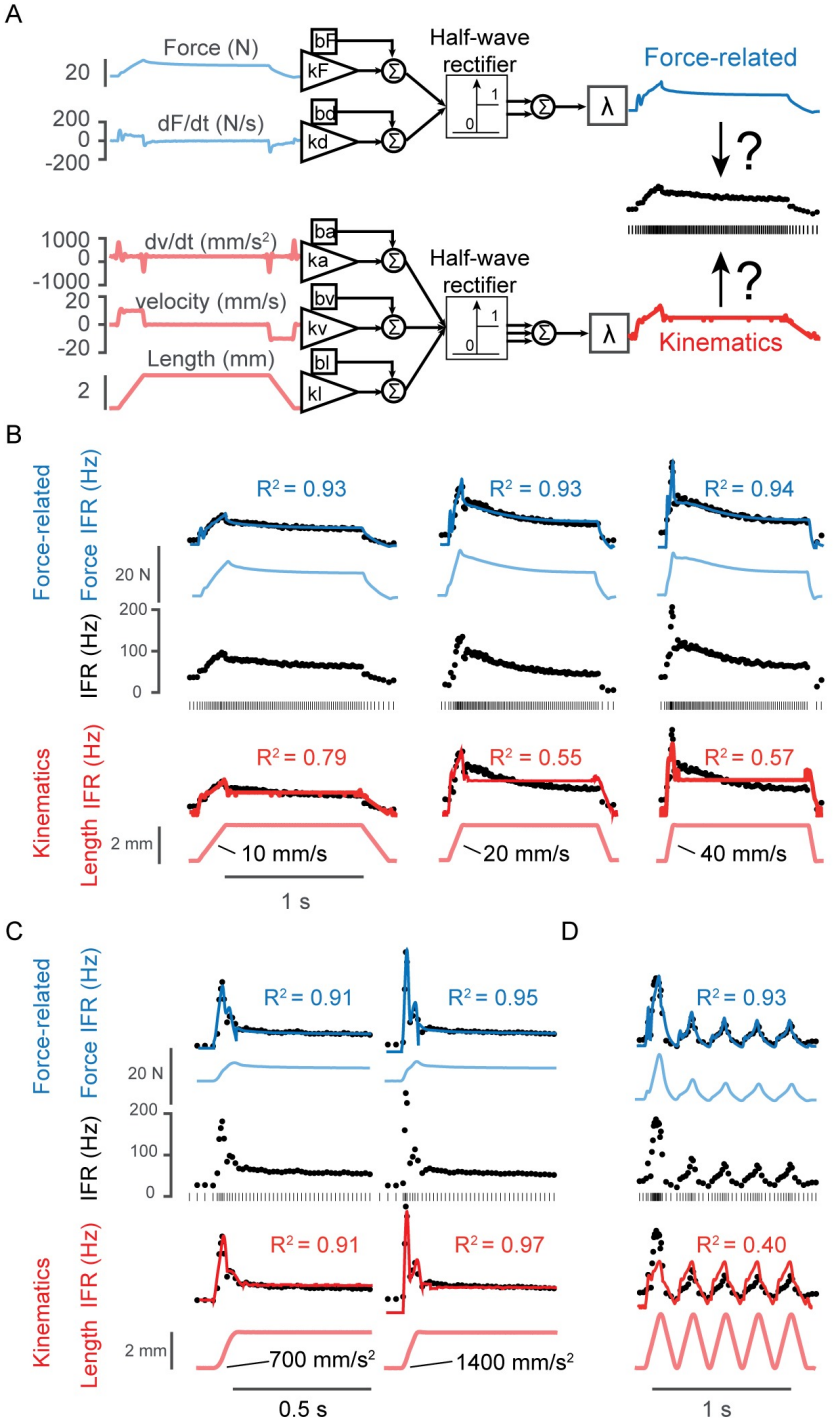
Reconstruction of muscle spindle firing rates by two candidate models. (A) Two main candidate models for predicting IFRs are shown: the force-related model used a combination of the force developed in the musculotendon and its first time-derivative as input (linearly combined, inputs and model prediction in blue); the length-related model used a linear combination of the muscle length, velocity, and acceleration (inputs and model prediction in red). The instantaneous firing rate of the afferent (black dots) was used to compute the error to optimize model parameters. (B) Three examples of muscle stretch with different stretch speed are shown (10, 20, and 40 mm/s). The stretch was sustained for 1s before being released. From top to bottom, the force-related model output (blue line) is overlaid on the instantaneous firing rate during the stretch; the recorded force (light blue) and the response of the afferent (black dots and raster) are shown directly below the force-related model output; the length-related model output (red line) is overlaid on the instantaneous firing rate and shown with the controlled musculotendon length (light red) below the response of the afferent. (C) The muscle was stretched to the same final length and at the same velocity, but with different initial accelerations (700 and 1400 mm/s^2^, respectively). (D) The muscle was stretched with sawtooth patterns, where stretches were not sustained but immediately released and repeated. In all cases, the force-related model fits were as good or better than the length-related model fits, as indicated by the R^2^ value. The examples shown are for afferents 4 (A-B, D) and 5 (C).

To test which musculotendon mechanical variables best reproduced transient, history-dependent muscle spindle IFRs, we developed 2 primary candidate models (Fig 3A). The first was a pseudo-linear combination of musculotendon force and dF/dt (Fig 3A, blue traces). The second was a pseudo-linear combination of musculotendon length, velocity, and acceleration (Fig 3A, red traces). In each model, only positive changes above a threshold value were retained and compared to recorded IFRs. To account for neuromechanical delays, a common lag was applied to each group of variables. For our initial analysis, we estimated parameters of each candidate model and computed the goodness of fit (coefficient of determination, R^2^) to recorded IFRs for each afferent on a per-stretch basis (Fig 3A). Here, we first present a few examples comparing force-related and length-related model fits to individual stretch responses. In a subsequent analysis, we provide more comprehensive comparisons of several models based on their ability to predict all stretches for each afferent using a single set of parameters.

### Force-related but not length-related variables reproduce rate relaxation in fast stretches

While both force- and length-related models could reproduce some features of muscle spindle IFRs, force-related variables better accounted for the details of the IFRs, especially at faster stretch velocities (Fig 3B). Both models qualitatively captured IFR features during a 10 mm/s stretch, with R^2^ of 0.93 and 0.79, respectively (Fig 3B, left column). As stretch velocity increased to 20 and 40 mm/s, the force-related model reproduced IFRs with high fidelity (Fig 3B, blue traces; R^2^ = 0.93, 0.94), whereas the length-related model had greatly reduced goodness of fit (Fig 3B, red traces; R^2^ = 0.55, 0.57).

Specifically, as the stretch velocity increased, rate relaxation increased during the hold period in concert with a decline in muscle force (Fig 3B, light blue traces). Whereas the dynamic response during the ramp stretches could be accounted for by either musculotendon dF/dt or velocity and acceleration (but underestimated by length-related variables for the 40 mm/s stretch; Fig 3B), the differences in musculotendon length and force were highly evident during the hold period. Over the hold period, muscle force (Fig 3B, light blue traces) decreased with a similar time course as the muscle spindle IFRs. As musculotendon length was constant, the length-related model first under-estimated, and then over-estimated IFRs during the hold (Fig 3B, red traces).

### Rate change in force, dF/dt, reproduces the initial burst and dynamic response to stretch

The initial burst IFRs increased when ramp accelerations were increased while keeping stretch length and velocity the same (Fig 3C), and was accounted for by both models. The initial burst IFRs were similarly accounted for by either dF/dt in the force-related model, or acceleration in the length-related model.

However, during a series of ramp-release muscle stretches, the dynamic response was better reproduced in the force-related vs. length-related model (Fig 3D). The differences in musculotendon force and length during repeated stretch and release, or “sawtooth” perturbations, were most evident when comparing the first stretch and release to the subsequent ones (Fig 3D, blue vs red traces). In an example afferent, musculotendon length changes were the same across all five stretches, whereas musculotendon force and muscle spindle IFRs were greater in the first stretch (Fig 3D), resulting in much better goodness of fit in the force-related versus length-related model.

### Muscle fascicle length-related variables do not reproduce muscle spindle Ia IFRs

**Fig 4:**
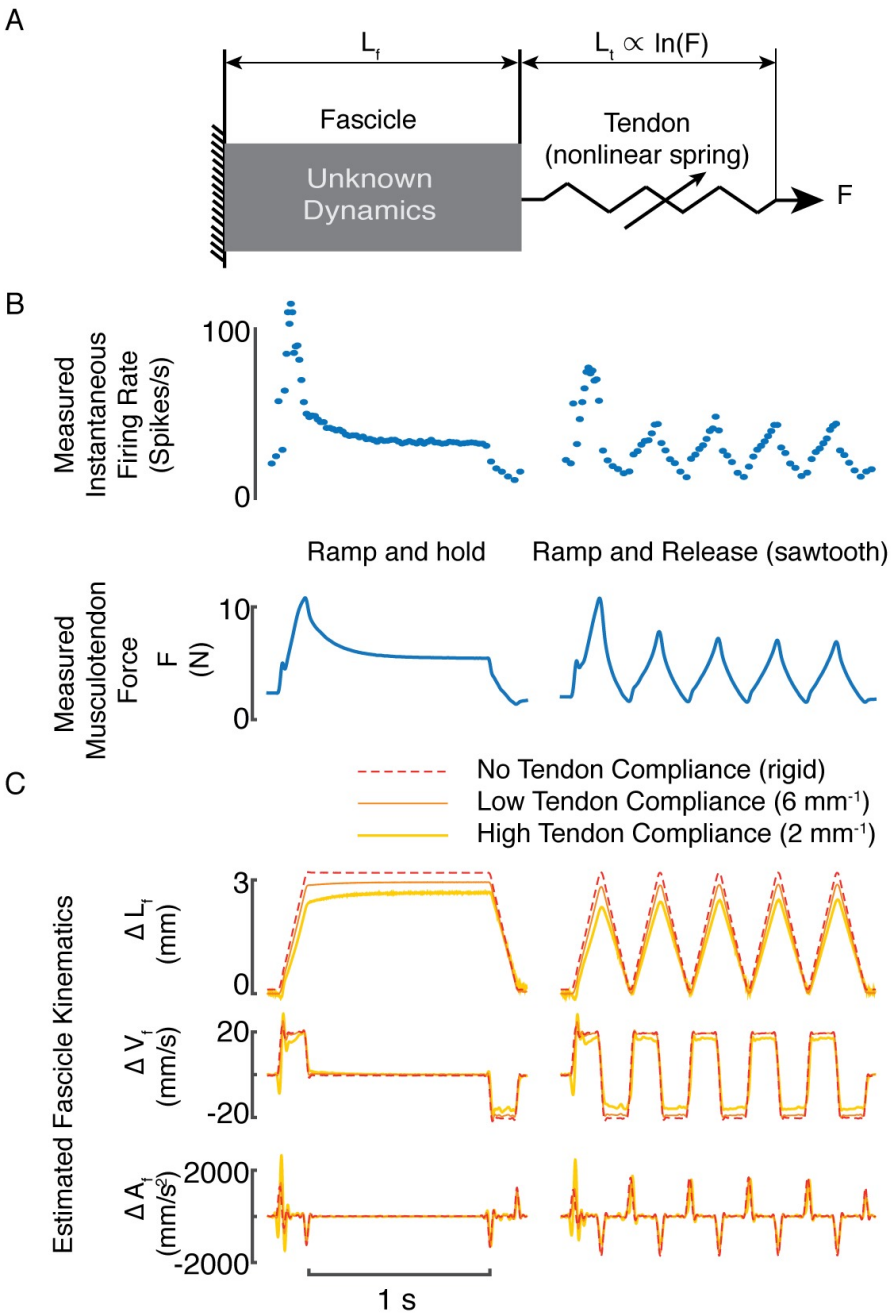
Estimated muscle fascicle length. (A) The Achilles tendon was assumed to be arranged in series with the triceps surae muscle fascicles. The measured musculotendon force was used to estimate tendon elongation. Muscle fascicle length was found by subtracting estimated tendon length from measured musculotendon length. A rigid tendon assumes changes in muscle fascicle length are equal to measured changes in musculotendon length. (B) Recorded IFR and measured musculotendon force in response to ramp-and-hold (left) and ramp-and-release stretches. Musculotendon force was used to compute estimated tendon elongation. (C) Example of estimated muscle fascicle length-related variables for ramp-and-hold stretch (left) and repeated ramp-and-release stretch (right). Top row is measured change in musculotendon length (red dashed trace) and two estimates of change in muscle fiber length (high tendon compliance = 2 mm^-1^: yellow; low tendon compliance = 6 mm^-1^: orange). The second and third rows are velocity and acceleration estimates, respectively, using the same coloring convention as for length.

To rule out the hypothesis that muscle spindles encode muscle fascicle and not musculotendon length-related variables, we refined our length-related model to include the effects of tendon elasticity. The inclusion of a tendon model allowed us to compare predicted IFRs based on musculotendon versus muscle fascicle length. We estimated muscle fascicle length changes by subtracting the stretch of the modeled tendon from recorded musculotendon length (Fig 4A). We did not explicitly consider pinnation angle; assuming a constant pinnation angle of the fascicles through the range of stretches would scale the length change by a constant factor and would not change our results. Tendon stretch was estimated by applying musculotendon force (Fig 4B) to an exponential model of Achilles’ tendon stiffness, based on the linear increase in tendon stiffness with muscle force shown in anesthetized cat tendon [35]. Based on two values of tendon stiffness, we estimated muscle fascicle length, velocity, and acceleration [35, 36] (6 mm^-1^, 2 mm^-1^, representing upper and lower limits of stiffness, respectively). Models based on muscle fascicle length-related variables typically produced worse goodness of fit values than whole-musculotendon length-related variables (Fig 4C). Specifically, of 1185 stretch perturbations across 10 afferents, goodness of fit based on muscle fascicle length-related variables were only higher in 5.2% and 19.1% instances for the high and low tendon stiffness estimates, respectively. Over all afferents, mean R^2^ values (± standard deviation) did not improve when using length-related variables based on muscle fascicle estimates (0.75 ± 0.13, and 0.70 ± 0.18 for high vs low tendon stiffness, respectively) versus those recorded from the whole musculotendon unit (0.77±0.12).

### Force-related model parameters are consistent across stretch velocity in all recorded afferents

**Fig 5:**
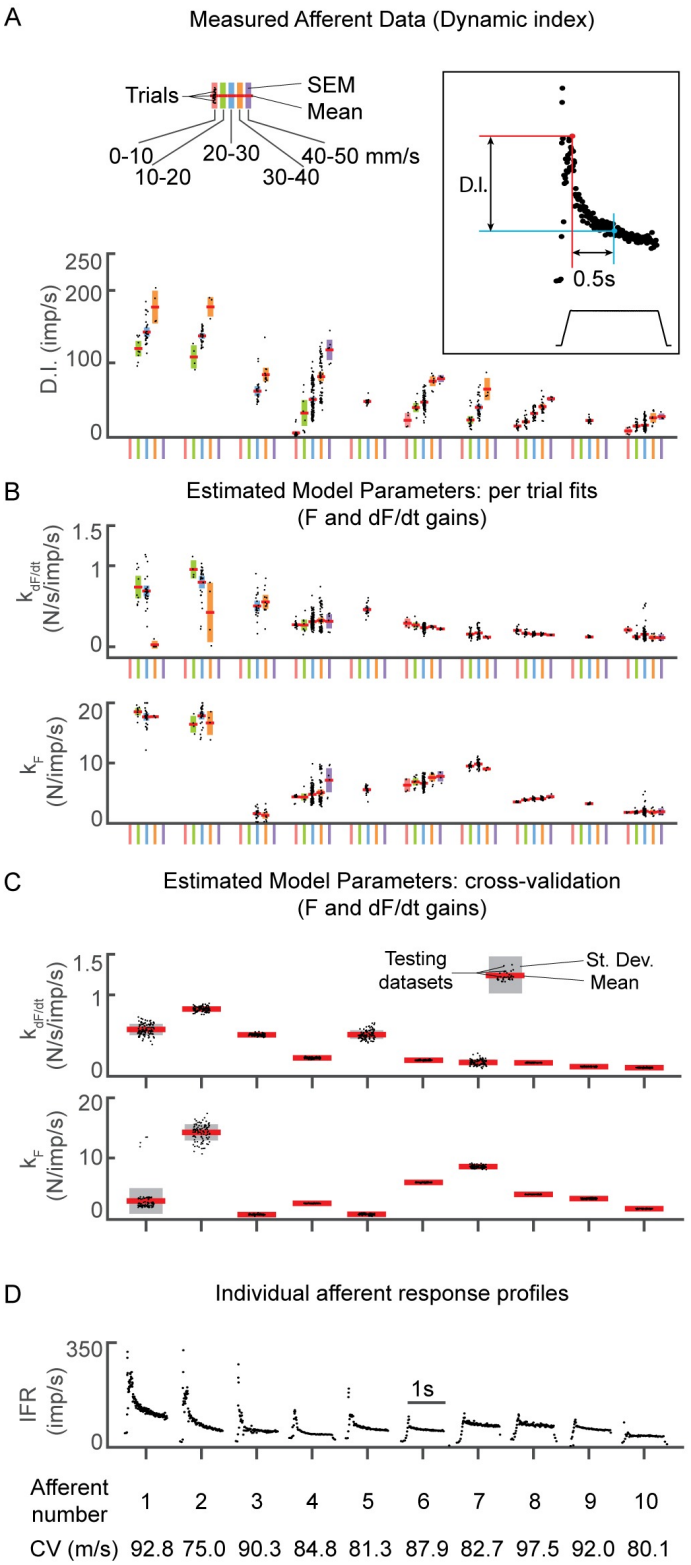
Single muscle spindle afferent statistics and model parameter estimates. (A) Dynamic index measured for each ramp-and-hold stretch trial for every afferent, organized in descending order from left to right. For the 10 afferents analyzed, stretch trials were separated based on the velocity (indicated by colors) imposed on the muscle for that trial (divided into 5 10 mm/s velocity bins ranging from 0–50 mm/s). Bins with fewer than 4 trials were excluded from this figure. Horizontal red lines represent the mean, the colored bars represent the standard error of the mean (SEM), and the black dots represent individual trial values. (B) In contrast to DI, force-related model weights were relatively constant with increasing stretch velocity. The color scheme is the same as in A. The upper plot is model weight on dF/dt and the lower plot is the model weight on force. (C) Force-related model weights and distributions for 100 randomized testing datasets (fitting one set of parameters for the entire dataset) for each afferent. For each afferent, a range of stretch perturbations (e.g. varying length, velocity, acceleration and stretch type) were included in the testing dataset. Red lines represent the means, grey bars represent standard deviations, and the black dots represent values for each testing dataset. As in B, model weights on dF/dt are shown in in the upper plot and model weights on force are shown in the lower plot. (D) Responses to a ramp-and-hold stretch at 3mm hold length, and 20mm/s stretch velocity for each afferent. Bottom row indicates conduction velocity for each afferent included in this analysis.

We used dynamic index (DI) to characterize the variations in dynamic sensitivity of the 10 analyzed muscle spindles to muscle stretch (Fig 5A). DI is a classical metric in which the IFR during the hold phase [10], measured 0.5 s after the end of the ramp phase, is subtracted from peak IFR at the end of the ramp phase (Fig 5A). Higher DI values characterize muscle spindles exhibiting relatively large initial bursts and dynamic response; these are typically attributed to large contributions to firing rates from terminals on bag 1 intrafusal fibers (e.g. Fig 3C). More dynamic afferents are also characterized by increasing DI with stretch velocity [10] (Fig 5A, left: colored bars). Lower DI values describe muscle spindles that respond less to ramp stretches, but maintain higher firing rates during the hold period (e.g. Fig 3B); these are typically attributed to large contributions to firing rates from terminals on bag2 and chain intrafusal fibers.

The recorded muscle spindle Ia afferents spanned the range from highly dynamic to static (Fig 5A, left to right). Where the datasets allowed, we calculated DI across stretch velocities (4–50 mm/s). As expected, for each afferent with trials at different stretch velocities, DI increased with stretch velocity (Fig 5A). Four afferents exhibited mean DIs of larger than 50 imp/s for 20mm/s stretch velocity (Fig 5A, blue error bars, afferent numbers 1–4; conduction velocities shown in Fig 4D), the only stretch velocity tested in every afferent. Two afferents exhibited mean DIs less than 30 imp/s at 20mm/s stretch velocity, which was unexpected given their conduction velocities (Fig 5A, blue error bars, afferent numbers 9–10; conduction velocities shown in Fig 4D). The remainder of afferents exhibited mean DIs between 30 and 50 imp/s at 20mm/s stretch velocity (Fig 5A, blue error bars, afferent numbers 5–8; conduction velocities shown in Fig 4D). A direct comparison of our DI measurements to the original measures of Matthews [10] could not be made because our estimates of DI were computed based on 3mm stretches, a lower amplitude than used by Matthews [10].

In contrast to DI, force-related model parameters for each afferent were similar across stretch velocities (Fig 5B). Weights for both dF/dt and force (k_dF_ and k_F_) were relatively consistent across trials (Fig 5B vs. 5A, colored blocks for each afferent) and were highest in the most dynamic afferents (Fig 5B, left).

### The most likely model of transient muscle spindle IFRs uses musculotendon force-related variables

We systematically assessed the ability of six candidate models to predict information contained in 10 muscle spindle IFR datasets. Briefly, for each afferent we randomly sorted the entire set of stretch perturbations into a training and testing dataset based on the number of available trials. For each model, we identified model parameters from the training dataset and then used them to predict IFRs in the testing dataset. To reduce potential data selection biases, this process was repeated on 100 randomized training and testing datasets.

A single set of force-related model parameters were identified that reproduced IFRs in each afferent across all stretch conditions (Fig 5C). As when fitting individual stretches, weights corresponding to dF/dt were typically highest in the most dynamic afferents (Fig 5C, left). Based on DI, weights corresponding to dF/dt for afferent 5 were higher than expected, but this afferent exhibited a relatively high initial burst, which is not captured by DI (same afferent as shown in Fig 3C).

**Fig 6:**
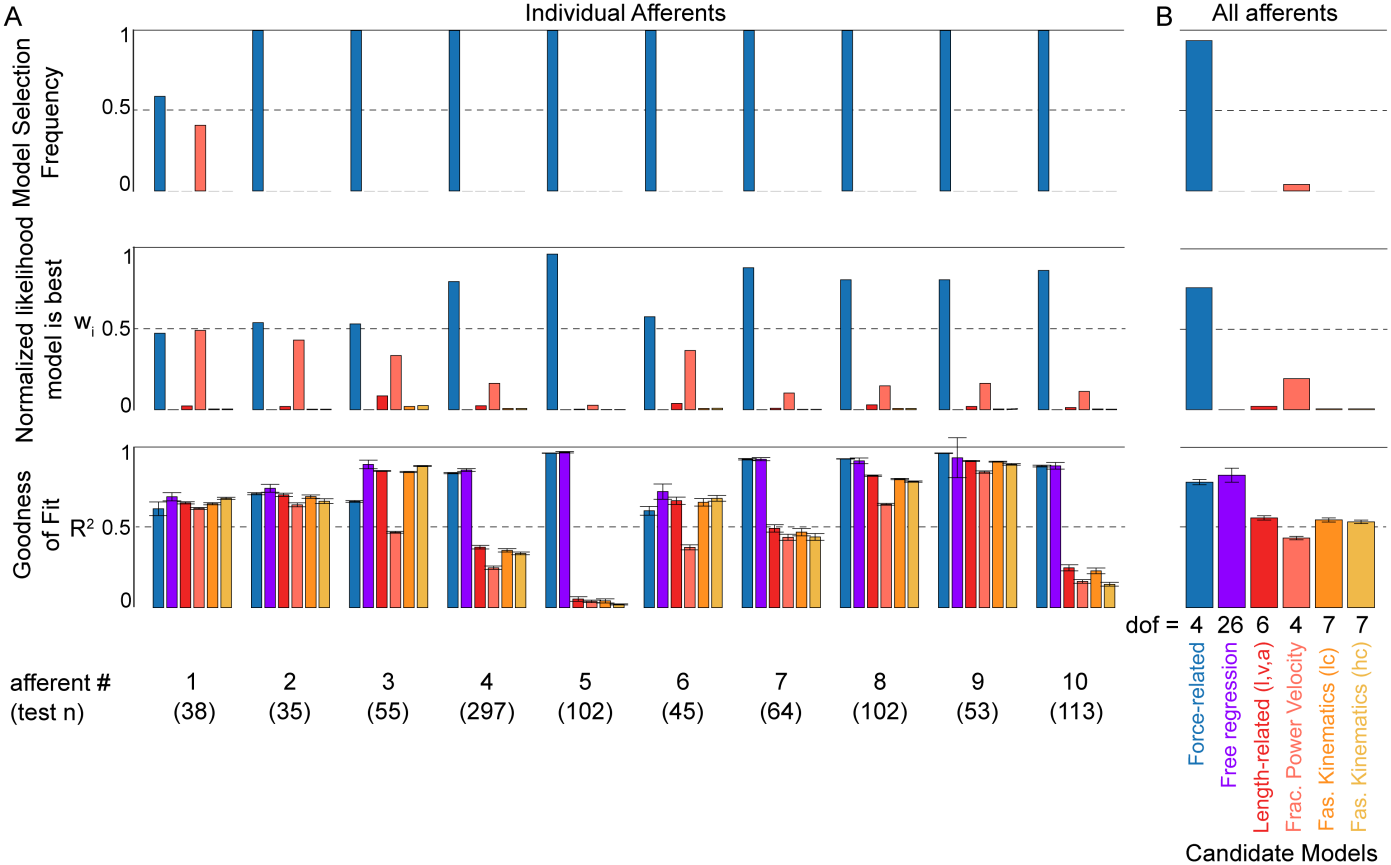
Comparison of candidate models’ abilities to predict muscle spindle Ia afferent IFRs. (A) Testing dataset model results for 10 afferents with 6 candidate models. The candidate models include one containing only force-related variables (force and its first time-derivative, dF/dt: blue), length-related variables (length, velocity, and acceleration: red), length-related variables with velocity raised to a fractional power (light red), muscle fiber length-related variable estimates (fiber length, velocity, and acceleration estimated with low and high tendon compliance: orange and light orange, respectively), and a free regression with all predictor variables from the other candidate models (purple). Top row: model selection frequency (number of times model was selected as the best candidate) for each model for 100 randomized testing datasets. Middle row: mean normalized likelihood (or Akaike weights, w_i_) that a given model is the best predictor of information in the IFRs, based on corrected Akaike Information Criterion (AICc) for regressions of the 100 randomized testing datasets. This is the relative weight of evidence in favor of a model being the best, given the set of candidate models. The sum of all 6 Akaike weights is equal to 1. Bottom row: mean R^2^ (coefficient of determination) calculated between afferent instantaneous firing rate and model prediction for 100 randomized testing datasets. Error bars represent 1 standard deviation. (B) Similar statistics to (A), but calculated for all 1000 randomized datasets (100 randomizations for 10 afferents). Top row: total model selection frequency for each model out of 1000 randomized datasets. Middle row: relative likelihood that each model is the best model in the set. This was calculated from the mean AICc values of all 10 afferents in (A). Bottom row: mean R^2^ across all 10 afferents in (A) for each model. Error bars represent 1 standard deviation.

In addition to the force-related model, the other 5 candidate models included combinations of musculotendon and muscle fascicle length-related variables. Candidate models included combinations of musculotendon length-related variables (Fig 6, red bars) described above (Fig 3A). Based on prior muscle spindle models, we also tested a model that allowed velocity to be raised to a fractional power [19, 37, 38] (Fig 6, pink bars). We included models using muscle fascicle length-related variables estimated using low and high tendon compliance (Fig 6, orange and yellow bars). Finally, we tested a combination of all predictor variables (Fig 6, purple bars).

We compared the likelihood that each candidate model best predicts the information contained in the recorded muscle spindle IFRs while accounting for over-fitting. We report the fraction of times each model was selected as the best candidate (Fig 6, top row) out of 100 randomized iterations of training and testing our models. Based on the Akaike information criterion (AICc, including a correction for finite sample sizes), we also computed the normalized likelihood, *w*_*i*_, that each model best reproduced the data, while taking into consideration the number of model parameters (Fig 6, middle row). For reference, goodness of fit was quantified using the coefficient of determination, R^2^ (Fig 6, bottom row).

The force-related model was selected as the best candidate for each afferent when analyzed individually. The force-related model was selected as the best model in most of the 100 iterations for each afferent (Fig 6A top row; selection frequency = 0.59 for afferent 1, selection frequency = 1.0 for every other afferent). The normalized likelihood of the force-related model as the best candidate was at least 0.5 for all afferents except one (Fig 6A middle row); the lowest values were found in afferents in which smaller data sets were available (Fig 6A; “test n” displayed below afferent number), indicating relatively stronger force-related model performance (when compared to length-related models) where more stretch conditions were applied to the muscle. The model containing all predictor variables yielded the highest R^2^ for every afferent (Fig 6B, bottom row purple bars). In 6 of the 10 muscle spindle afferents, mean R^2^ of the force-related model was much higher than the remaining models (Fig 6A, bottom row blue bars).

The force-related model was also selected as the best candidate for the entire set of muscle spindle afferents. The force-related model was always the best predictor of information in the IFRs of the testing dataset (Fig 6B). As in the individual afferent analysis, the force-related model was the most frequently-selected model across the population of afferents (Fig 6B, top row; selection frequency = 0.959). When considering the entire population of afferents, the normalized likelihood (*w*_i_) of a model being the best within the set of candidates was greatest for the force-related model because it used fewer parameters than the model containing all predictor variables, but still predicted most of the information contained in the IFRs (Fig 6B, middle row; w_force_ = 0.77). Goodness of fit was highest in the model containing all 11 predictor variables (Fig 6B, bottom row, purple bar; R^2^ = 0.86 ± 0.046). Across all other models, mean R^2^ across afferents was highest for the force-related model (Fig 6B blue, R^2^ = 0.82 ± 0.017 for force-related model; red, R^2^ = 0.58 ± 0.014 for length-related model); R^2^ < 0.58 for other length-related models).

## Discussion

To our knowledge, this is the first demonstration of muscle spindle Ia afferents firing in direct proportion to muscle force and the first time-derivative of force, dF/dt, when passive, i.e. electrically-quiescent, muscles are stretched. While qualitative comparisons have been made previously between intrafusal tension transients and muscle spindle receptor potentials [34], this is the first quantitative description of a relationship between the spindle and muscle force response to stretch. The whole-muscle forces we recorded closely resembled transient force properties in stretched isolated muscle fibers that are attributed to calcium-dependent muscle cross-bridge interactions, including short-range stiffness and other history-dependent properties, despite the absence of measurable electrical potentials in the muscle. Our analyses show that muscle force and dF/dt parsimoniously define a unique transformation between muscle mechanical events and muscle spindle firing rates during passive muscle stretch. Therefore, consideration of history-dependent muscle forces could improve current muscle spindle models, which do not predict history dependent firing rates. Further, muscle spindles exhibiting a more dynamic response had higher sensitivity to dF/dt, while more static muscle spindles were primarily sensitive to muscle force. The initial bursts at the onset of stretch were proportional to dF/dt and acceleration, and likely play a functional role in sensorimotor responses to external perturbations, particularly during posture and balance [2, 4, 6, 39, 40]. The transient spiking features we analyzed were similar to those reported in the classic literature in muscle spindles [41]; our unique approach was to dissociate muscle force and length by recording a large, diverse set of muscles stretch conditions from each afferent, including variations in muscle acceleration that was independent of velocity. Further work is warranted to test whether encoding of muscle force will extend to active conditions, when fusimotor and skeletofusimotor motoneurons are firing, and its potential to explain load sensitivity and the non-unique relationships of muscle spindle firing rates to muscle length shown recently in humans [42, 43].

Our experimental measures of whole muscle force were likely correlated to intrafusal muscle fiber forces within the encoding regions of muscle spindles arising from muscle cross-bridge mechanics. It is unlikely that the forces from intrafusal fibers made a significant contribution to the recorded force or, conversely, that the extrafusal force had a significant contribution to the intrafusal fiber forces. Our assumption is that intrafusal and extrafusal forces exhibited similar history-dependent effects when stretched. Thus, we used the whole muscle force as a surrogate for intrafusal force, which is reasonable under anesthetized conditions, and consistent with our ability to predict fine details of muscle spindle IFR based on extrafusal muscle force. Our whole muscle forces during stretch closely resembled forces from experiments of single, permeabilized muscle fibers, suggesting little contribution of non-contractile tissue to whole muscle force in this experimental condition. Similar to our whole muscle recordings, isolated muscle fiber forces exhibit short-range stiffness when stretched after being held isometrically, characterized by a rapid rise in force at the onset of stretch, where the first time-derivative of force, dF/dt, increases transiently [27–29–2, 44, 45]. Both our whole muscle and isolated muscle fibers also exhibit history-dependent characteristic of dynamic response to ramp stretch and post-stretch rate relaxation. These history-dependent force transients are only present in isolated muscle fibers when attachment and detachment of muscle cross-bridges is possible due to the presence of low concentrations of Ca^2+^ [28, 29, 32]. Thus, even in passive, i.e. electrically-quiescent muscles, we see evidence of these transients in whole muscle force and dF/dt predict history-dependent muscle spindle initial bursts, dynamic response, and rate-relaxation after being held isometric, suggesting that muscle cross-bridge cycling is present at low levels. There may be differences that we did not account for due to the potential different myosin isoforms expressed in intrafusal and extrafusal muscle fiber [46]. Nevertheless, in the anesthetized conditions of our experiments, it seems reasonable to assume that whole-muscle force transients were similar to *in vivo* muscle fiber force, including intrafusal muscle fibers within muscle spindle sensory organs. Indeed, the initial burst of muscle spindle firing at the onset of muscle stretch has been described as “an intrafusal manifestation of the passive short-range stiffness of the extrafusal muscle” [23].

Our results show that muscle force-related variables define a unique transformation between muscle mechanical events and muscle spindle firing rates during stretch of passive muscle. Muscle force and its first time-derivative could reliably predict previously-observed history-dependent features of muscle spindle IFRs such as the initial burst, dynamic response, and rate relaxation that cannot be explained by muscle length-related variables. Moreover, a single set of model fit parameters produced high-fidelity predictions of muscle spindle IFRs across a wide range of stretch conditions. Our data quantitatively support the hypothesis that muscle spindles encode muscle fiber force [13, 23], which could not previously be tested due to technical limitations. Further, recordings of receptor potentials in muscle spindle encoding regions are qualitatively similar to the history-dependent transients in muscle force and dF/dt that we used to predict muscle spindle firing rates [34]. The role of anatomically-distinct encoding regions of the muscle spindle, i.e. bag1, bag2, chain regions [18, 47, 48] as well as rapidly- and slowly-adapting force sensitive ion channels [49–51] in encoding force-related information in muscle spindles remains to be fully explained, although contributions of distinct encoding sites to muscle spindle afferent firing rates have been demonstrated [52].

Our results suggest that adding muscle cross-bridge kinetics to computational models of muscle spindles would produce history-dependent muscle spindle firing rates. Sarcomere-level muscle models of cross-bridge kinetics can predict all of the history-dependent features of muscle fiber force described above, but generally require significant computation time [27–29]. Moreover, several muscle spindle models assume that force and dF/dt of intrafusal muscle fibers give rise to muscle spindle firing rates, but use more computationally-efficient phenomenological muscle models, e.g. Hill-type models, to estimate intrafusal muscle fiber force based on muscle length [19–21]. Hence, prior models do not reproduce the history-dependent features of muscle spindle IFRs that we studied. Computationally efficient simulation of muscle forces based on cross-bridge kinetics is thus critical for reproducing muscle spindle history-dependence, particularly sensorimotor responses to perturbations, where Hill-type muscle models lack necessary history-dependent properties to explain experimental results [53].

Muscle spindle subtypes may be characterized based on their individual sensitivities to force (k_F_) and the first time-derivative of force (k_dF/dt_). Classic measures of dynamic index are specific to stretch condition and only compare peak and steady-state firing rate during specific time windows of muscle ramp stretches. In contrast, constant parameters characterizing sensitivity to force and dF/dt take into account the entire time course of muscle spindle spiking activity. In our study, slowly-adapting, or “static” muscle spindles that had a low dynamic index lacked an initial burst and responded primarily to muscle force. Rapidly-adapting, or “dynamic” muscle spindles with a high dynamic index, exhibited initial bursts due to higher sensitivity to the transients in the first time-derivative of force, dF/dt, and were also sensitive to muscle force. While dynamic index varied with stretch velocity, sensitivity to force and dF/dt was invariant across different stretch conditions, making it a robust metric allowing comparisons across different stretch types. Further, the invariance of sensitivity to force and dF/dt within each afferent underscores the potential mechanistic relationship between muscle fiber forces and muscle spindle firing rates, providing a quantitative parameter of static and dynamic sensitivity. It remains to be seen whether the effects of alpha-gamma co-activation, which can enhance muscle spindle sensitivity in a subpopulation of afferents [54] can also be quantified in terms of sensitivity to force and dF/dt. Tuning the sensitivity of muscle spindle afferents could be a mechanism for tuning the net population firing rate arising from muscle spindle afferents during external perturbation.

Muscle spindle initial bursts that scale with stretch acceleration have been shown previously and can be explained by sensitivity to the first peak in dF/dt. Initial bursts have been observed in both acute animal experiments and in human microneurography in awake participants [10, 15, 22, 43, 54, 55] and have been largely attributed to stretch acceleration [16, 17, 38]. In our experiments, the initial burst scaled equally well to initial stretch acceleration or the peak in dF/dt, although later transients were only described by dF/dt. Although the number of spikes may be low in animal experiments using relatively fast stretches, in human microneurography studies using slow stretch velocity, as many as 20 spikes have been observed [55, 56]. Moreover, visual inspection of joint torque traces presented by Cordo and colleagues [55] reveal similarities in muscle spindle initial bursts and joint torque transients.

Muscle spindle initial bursts are likely critical in generating rapid and predictive corrective sensorimotor responses to external perturbation. Our prior experimental work demonstrates that corrective muscle activity elicited in long-latency responses to standing balance perturbation exhibit an initial burst of activity that scales with perturbation acceleration [2–6]. Moreover, the initial burst in muscle activity is lost in animals with large fiber sensory neuropathy in which muscle spindle Ia afferents are destroyed; these animals also exhibit balance impairments [6, 7]. As muscles are near isometric during quiet standing [57], muscle spindles within those muscles are likely to exhibit initial bursts at the onset of a perturbation due to transients in muscle force and dF/dt. Indeed, our simulation studies demonstrate that short-range stiffness is necessary to account for measured joint torque-angle relationships during perturbation to standing [58]. Further, theoretical studies show that delayed feedback based on acceleration or force can preemptively evoke a corrective response before large displacements occur, acting as a predictive controller [59]. It is currently unclear whether the sensory feedback required for these postural responses arises from active or passive muscles, or both. Though our study does not directly test the generalization of force encoding to active muscle, there is evidence that initial bursts persist with gamma activation [60] and that acceleration-dependent sensorimotor bursts occur in active muscle during postural perturbations [2–4, 6, 61]. Thus, the initial burst of muscle activity that scales with acceleration is likely due to muscle spindle initial bursts and may explain why peak activity of postural muscles occurs prior to peak displacement [2, 57].

We speculate that force encoding in muscle spindles could underlie other non-unique relationships observed between joint motion and perceived limb position, and between muscle force generation and muscle spindle firing rates [62]. Muscle spindles play a role in both conscious and unconscious motor control [62]. To a degree, the CNS is likely capable of compensating for any shortcomings of the spindle as a length transducer with contributions from other sensory modalities (e.g. cutaneous receptors, which have an important contribution to proprioception). However, there are important circumstances in which history-dependent muscle spindle behavior such as thixotropy can impair the position sense provided in part from muscle spindles. When the limbs are passively manipulated, perceived joint position exhibits similar history-dependence to muscle spindle firing, causing an illusion in joint angle if the muscle is first stretched versus activated prior to stretch [24, 26]. Muscle spindle sensory information is distinct from that provided by Golgi tendon organs, which encode a combination of force and the first derivative of muscle *contractile* force due to efferent drive to muscles [63], but are generally silent during passive stretch and lack an initial burst [64]. Recent work demonstrates that muscle spindle firing is not uniquely related to muscle length during active muscle contractions; instead, the rate of firing depends on magnitude and direction of external load, not joint kinematics [43]. Human muscle spindle primary afferents have also been shown to fire distinctly during different stages of learning regardless of similar movement kinematics [42]. Another recent study showed that firing rates in human muscle spindle primary afferents in response to sinusoidal changes in ankle angle are only predicted by muscle length at steady-state and not during transient firing at the onset of imposed movements [65]. Our findings predict that history-dependent muscle forces, which are difficult to measure *in vivo*, would predict steady-state as well as transient firing in muscle spindle afferents. During alpha-gamma coactivation, such as during voluntary muscle contractions [43, 54] and perceived postural threat [66], intrafusal muscle spindle fibers that lie in parallel with extrafusal muscle fibers are also activated; we speculate that this increase in muscle activity would increase the sensitivity of the muscle spindle to errors in muscle force and rate change in force due to unanticipated environmental interactions, relative to the anesthetized conditions here that lack efferent drive. Indeed, alpha-gamma co-activation has been shown to modulate muscle spindle primary afferent dynamic sensitivity based on loading of the spindle-bearing muscle in response to external perturbations [54]. This interpretation is consistent with the idea that muscle spindles inform internal models for movement, providing estimates of sensory error based on efference copy [67]. However, much further study is necessary to test whether the encoding of whole-muscle force in passive muscle spindles generalize to conditions involving active muscle contraction. As muscle force is a good proxy for muscle length in many conditions, we hypothesize that that the biophysical transformation from passive muscle stretch to muscle spindle firing is based on muscle force transients but potentially interpreted perceptually in terms of joint position and velocity.

## Materials and methods

### Ethics statement

In accordance with French legislation, the animal facility, experimental room, and investigators had valid licenses (75-789-R) to perform experiments on live vertebrates delivered by the Direction des Services Vétérinaires (Préfecture de Police, Paris, France). Animals in this study were anesthetized with sodium pentobarbital given intravenously, and monitored continuously. Animals were sacrificed at the end of the experiments with a large bolus of sodium pentobarbital also given intravenously.

### Data collection

We collected data from muscle spindle Ia afferents in response to stretch perturbations of the isolated triceps surae musculotendon in anesthetized cats. Experiments were performed on 5 adult cats (2.8–3.3 kg) deeply anesthetized with sodium pentobarbital (Pentobarbital; Sanofi Recherche, Montpellier, France). Anesthesia was induced with an initial intraperitoneal injection (45 mg/kg). Animals were deeply anesthetized, with the level of anesthesia constantly assessed by monitoring the stability of the heart rate, blood pressure (measured through a carotid catheter), the maintenance of myotic pupils, and the absence of nociceptive reflex. It was supplemented whenever necessary (usually every 2 h) by intravenous injections (3–6 mg/kg) of pentobarbital. The anesthesia was deep enough to prevent any pain as indicated by the fact that noxious stimuli (induced by pinching the ear or the foot paw) did not elicit any heart acceleration or blood pressure changes. Animals were paralyzed with Pancuronium Bromide (Pavulon; Organon, Puteaux, France) at a rate of 0.4 mg/h and artificially ventilated (end-tidal P_CO2_ maintained at ∼4%). This allowed us to perform a bilateral pneumothorax in order to prevent movements of the rib cage during the recordings. At the onset of experiment, amoxicillin (500 mg; Clamoxyl; Merieux, Marcy l'Etoile, France) and methylprenidsolone (5 mg; Solu-Medrol; Pfizer, New York, NY) were given subcutaneously to prevent the risk of infection and edema, respectively. The central temperature was kept at 38°C with the help of a thermo-controlled blanket. Blood pressure was maintained above 90 mmHg by perfusion of a 4% glucose solution containing NaHCO_3_ (1%) and gelatin (14%; Plasmagel, Roger Bellon Laboratories, Neuilly, France) at a rate of 3–12 ml/h. A catheter allowed evacuation of urine from the bladder. At the end of the experiments, animals were given a lethal intravenous injection of pentobarbital (250 mg).

The dorsal aspect of lumbar segments (L1-L6) was exposed to allow electrode access to the dorsal columns. The triceps nerve was dissected without alteration of its continuity with the muscle and mounted on a bipolar stimulation electrode. The Achilles tendon was detached from the calcaneus and the isolated bone fragment attached to the tendon was affixed to a muscle puller (Aurora 310B LR, Aurora, ON, Canada) to control the elongation of the attached muscle and to measure the force developed at the tendon. The initial length of the triceps was set to maintain a low level of resting tension (typically 0.1 N) but could be adjusted to reach a low resting firing of the afferents. A pool filled with mineral oil heated at 38°C was made around the triceps surae and its nerve.

A sharp microelectrode (KCl 3M, Resistance 11.5–20 MΩ) was driven into the dorsal horn of the spinal cord at the location of the largest afferent volley upon electrical stimulation of the triceps nerve. The high electrical resistance of the electrode allowed us to isolate a single afferent that displayed an all-or-none spike in response to nerve stimulation. Identified afferents were classified as Ia based on axonal conduction velocity ≥ 74 m/s and on response to the rising phase of a small stretch. To facilitate spike detection, voltage recordings were high-pass filtered at 500 Hz to remove drift. Recordings lasted as long as the signal-to-noise ratio allowed us to differentiate spikes from background noise.

Musculotendon length, velocity, and tension were sampled at 2 kHz and used to calculate acceleration and the first time-derivative of tension. Low-pass filters were applied to velocity (40 Hz), acceleration (40 Hz), Force (50 Hz), and dF/dt (100 Hz).

Custom musculotendon stretch profiles were designed in Spike2 software to dissociate the effects of velocity and acceleration on the response of the afferent. Multiple stretch perturbation waveforms ranged from 1–4 mm in peak length, 4–50 mm/s in peak velocity, and 200–3500 mm/s^2^ in peak acceleration. Rest periods of at least 5 seconds were included between all perturbations (in repeated ramp-release stretches, rest periods of at least 5 seconds were used in between sets of 5 stretches).

### Sample size

No explicit *a priori* power analysis was performed to determine sample size. Experiments were performed over the course of 5 months. At the conclusion of this period, data were screened for quality and it was determined that the available sample of recorded afferents was commensurate with a previous study with similar methodology [37].

### Data inclusion and exclusion

Analyses treated individual recorded afferents and individual recorded stretch trials as biological replicates and as technical replicates, respectively. To ensure sufficient information for statistical measures, we required that stretch trials have at least 50 recorded action potentials in order to be included in statistical analyses. Stretch trials with low signal to noise ratio based on visual inspection were excluded. To ensure that each individual recorded afferent had sufficient observations to enable cross-validation during model fitting (below), we required that afferents have at least 40 acceptable stretch trials. This guaranteed at least 30 observations of testing data for each afferent. These criteria yielded suitable datasets for 10 individual afferents. Data available from the Dryad Digital Repository: http://dx.doi.org/10.5061/dryad.pd40m [68].

### Initial burst correlations

For correlating the peak initial burst amplitudes with preceding peaks in transient force- and length-related variables (dF/dt and acceleration, respectively), we performed simple least squares linear regressions in Matlab that found a linear function relating peak dF/dt or acceleration to initial burst amplitude,

$$\hat{y}=b_{1}x+b_{2}$$

where $\hat{y}$ is the estimated initial burst amplitude predicted by x (either peak dF/dt or acceleration). To measure the amount of variance in peak initial burst amplitude explained by peak dF/dt and acceleration, we computed the coefficient of determination

$$R^{2}=1-\frac{\sum_{i}{\left(y_{i}-{\hat{y}}_{i}\right)}^{2}}{\sum_{i}{\left(y_{i}-\bar{y}\right)}^{2}}$$

where *y* is the recorded value of initial burst amplitude, $\hat{y}$ is the predicted initial burst amplitude, $\bar{y}$ is the arithmetic mean of the recorded initial burst amplitudes, and *i* denotes the *i*th data point.

### Candidate models

For testing the information encoded by muscle spindle Ia afferent IFRs, we developed 6 candidate models. Candidate models were pseudo-linear in that they used weighted sum of measured variables subjected to a half-wave rectification to account for the unidirectional effects of muscle stretch on muscle spindle spiking behavior (Fig 2A). The primary candidate models consisted of linear combinations of musculotendon length-related and force-related variables (Fig 2). Based on previous observations, we tested a model that allowed for muscle velocity to be raised to a fractional power [19, 37, 38]. In addition, we also tested the efficacy of length-related variables based on estimated muscle fascicle length using both high and low tendon stiffness values (Fig 5).

For certain afferents, force and dF/dt components were modeled as competing influences on the IFR, as done previously by Lin and Crago [20]. At time points where simultaneous contributions of force and dF/dt were significant, we used an iterative process to set the values of the smaller-magnitude component to zero. The two components were then summed as in the original force-related model. We used the competing model if the mean R^2^ achieved across all observations was higher than for the original model. This was the case in 4 out of 10 afferents (1, 2, 3, and 6), which also had the largest contributions of dF/dt to the IFR (see Fig 6B and 6C). Although the mechanism of the competition is not clear, it could result if the effects of force and dF/dt components arise from different branches of the muscle spindle primary afferent endings. Dynamic sensitivity of the muscle spindles is controlled by terminals innervating the bag1 intrafusal fiber, whereas the static response is controlled by the terminals innervating the bag2 and chain intrafusal fibers [52].

### Fascicle length-related variable estimates

To estimate muscle fascicle length-related variables, we assumed an exponential elastic tendon, based on experiments by Rack and Westbury [35], to be serially aligned with the muscle fascicle such that the force was equal in the fascicles and the tendon (Fig 4). Using measured musculotendon force, we estimated tendon elongation during stretch and subtracted it from measured musculotendon elongation to estimate fascicle length. We assumed that the pinnation angle of the muscle fascicles remained constant throughout the range of stretches applied to the musculotendon in these experiments. Because a constant pinnation angle would only decrease the fascicle lengths by a constant factor, and because this factor would be eliminated when the muscle fascicle length-related variables were scaled and offset in the model, we ignored the effects of pinnation altogether. We calculated fascicle velocity and acceleration using numerical differentiation of estimated fascicle length.

### Time lags and parameter search limits

In order to determine appropriate time lags and parameter search limits for each candidate model, we fit the estimated IFR for each of the 6 candidate models to the IFR of the afferent for a selection of stretch perturbations applied to the muscle. The candidate model parameters, consisting of a weight (*k*_*i*_) and offset (*b*_*i*_) for each force-related or length-related variable included in the sum, were found via least-squares regression using Matlab’s optimization toolbox (*fmincon*.*m*) and custom scripts. A lumped neuromechanical delay (*λ*_*j*_) was determined by shifting the timestamp of the muscle variables (e.g. length, force) forward relative to the IFR data to be fit. The form of the models was:

$$IFR_{j}\left(t\right)=\left(\overset{n}{\underset{i=1}{\sum }}k_{i}\cdot \left(\left\lfloor x_{i}\left(t-\lambda_{j}\right)\right\rfloor +b_{i}\right)\right)+e\left(t\right)$$

where the IFR of the jth model was estimated by a sum of n force- or length-related variables, each of which was temporally shifted by a neuromechanical delay, *λ*_*j*_ (held constant for each model for a given trial), offset by a single value, *b*_*i*_, and scaled by a gain, *k*_*i*_. ⌊ ⌋ denote positive values of the argument. Error, *e*(*t*), was minimized by finding the set of parameters for each model that minimizes a measure related to *e*(*t*)^2^ (see Data Fitting). In all, the optimization swept through 16 values of *λ*_*j*_, in 1 ms increments from 0 to 15 ms. Then, the lag value for each trial which yielded the most explained variance (highest R^2^ value) were averaged to determine a single lag value for each afferent. This value was also used later for prediction and cross-validation analyses (Fig 5B). The weights and offsets (i.e. *k*_*i*_ and *b*_*i*_) found in this analysis were used to appropriately restrict the parameter search in the prediction and cross-validation analyses of the complete afferent datasets. No statistical tests were performed on the parameters estimated from this analysis.

### Cross-validation

We used an iterative cross-validation procedure to robustly estimate the fit of each model to recorded IFR data of each afferent. For each model and each afferent, we performed cross-validation as follows. All available stretch perturbations were randomly divided into training (≈75%) and test (≈25%) sets. Using the training dataset, model parameters were identified that minimized the cost function

$$\;J=\;\frac{\sum_{i}{\left(y_{i}-f_{i}\right)}^{2}}{\sum_{i}{\left(y_{i}-\bar{y}\right)}^{2}}=\;\frac{SSE}{SSM}$$

where the numerator represents the sum of squared errors (SSE) between the afferent IFR (*y*) and the model estimate of IFR (*f*), and the denominator represents the total sum of squares of the IFR about its mean (SSM). Model fit (R^2^) was then assessed using the test IFR dataset. Mean ± SD R^2^ values were calculated over a set of 100 iterations of this cross-validation procedure for each model and each afferent.

### Model selection

We used the approach of Burnham and Anderson [69] to determine which model among our candidate models performed best at predicting recorded muscle spindle spike data. Test set AICc (Akaike Information Criterion, corrected for finite sample sizes) was calculated for each model for each afferent as:

$$AICc=2\left(k-\text{ln}\left(\hat{L}\right)-\frac{k\left(k+1\right)}{n-k-1}\right)$$

where k is the number of estimable parameters in the model, $\hat{L}$ is the maximum value of the likelihood function for the model (here, estimated as J^-1^ of the test set), and n is the number of recorded stretch trials in the test set. Differences in AICc between models for each afferent were calculated as:

$$\Delta_{j}=AICc_{j}-\text{min}\left\{AICc_{1}\ldots AICc_{6}\right\}$$

where Δ_*j*_ is the difference in AICc between the jth model and the minimum AICc from the set of candidates. AICc differences were used to calculate a set of Akaike weights:

$$w_{j}=\frac{\text{exp}\left(-\frac{1}{2}\Delta_{j}\right)}{\sum_{r=1}^{6}\text{exp}\left(-\frac{1}{2}\Delta_{r}\right)}$$

where *w*_*j*_ is the normalized likelihood of the jth model being the best in the set.

To assess the models’ abilities to predict the afferent population IFRs, we used similar statistical methods as for the individual afferents. To measure goodness of fit for the entire afferent population, we took the average model R^2^ values for each afferent from the 100 optimization procedures and averaged them together, resulting in a single R^2^ value for the population (Fig 5A). Then, we used a similar averaging procedure to obtain a single AICc value for each model. From these values, we calculated *w*_*j*_ for the entire population as described above.

## References

[1] Macpherson JM, Horak FB. Posture In: Kandel ER, Schwartz JH, Jessell TM, Siegelbaum SA, Hudspeth AJ, editors. Principles of Neural Science. 5th Edition ed: McGraw-Hill Education / Medical; 2013.

[2] Welch TD, Ting LH. A feedback model reproduces muscle activity during human postural responses to support-surface translations. Journal of Neurophysiology. 2008;99(2):1032–8. doi: 10.1152/jn.01110.2007 18094102 10.1152/jn.01110.2007

[3] Welch TD, Ting LH. A feedback model explains the differential scaling of human postural responses to perturbation acceleration and velocity. Journal of Neurophysiology. 2009;101(6):3294–309. doi: 10.1152/jn.90775.2008 19357335 10.1152/jn.90775.2008 PMC2694108

[4] Safavynia SA, Ting LH. Long-latency muscle activity reflects continuous, delayed sensorimotor feedback of task-level and not joint-level error. Journal of Neurophysiology. 2013;110(6):1278–90. doi: 10.1152/jn.00609.2012 23803325 10.1152/jn.00609.2012 PMC3763153

[5] Safavynia SA, Ting LH. Sensorimotor feedback based on task-relevant error robustly predicts temporal recruitment and multidirectional tuning of muscle synergies. Journal of Neurophysiology. 2013;109(1):31–45. doi: 10.1152/jn.00684.2012 23100133 10.1152/jn.00684.2012 PMC3545166

[6] Lockhart DB, Ting LH. Optimal sensorimotor transformations for balance. Nature Neuroscience. 2007;10(10):1329–36. doi: 10.1038/nn1986 17873869 10.1038/nn1986

[7] Stapley PJ, Ting LH, Hulliger M, Macpherson JM. Automatic postural responses are delayed by pyridoxine-induced somatosensory loss. The Journal of Neuroscience. 2002;22(14):5803–7. 12122040 10.1523/JNEUROSCI.22-14-05803.2002 PMC6757909

[8] Chiel HJ, Ting LH, Ekeberg Ö, Hartmann MJ. The brain in its body: motor control and sensing in a biomechanical context. Journal of Neuroscience. 2009;29(41):12807–14. doi: 10.1523/JNEUROSCI.3338-09.2009 19828793 10.1523/JNEUROSCI.3338-09.2009 PMC2794418

[9] Ting LH, Chvatal SA, Safavynia SA, Lucas McKay J. Review and perspective: neuromechanical considerations for predicting muscle activation patterns for movement. International Journal for Numerical Methods in Biomedical Engineering. 2012;28(10):1003–14. doi: 10.1002/cnm.2485 23027631 10.1002/cnm.2485 PMC4121429

[10] Matthews P. The response of de‐efferented muscle spindle receptors to stretching at different velocities. The Journal of Physiology. 1963;168(3):660–78. 14067950 10.1113/jphysiol.1963.sp007214 PMC1359446

[11] Matthews P, Stein R. The sensitivity of muscle spindle afferents to small sinusoidal changes of length. The Journal of Physiology. 1969;200(3):723 4237132 10.1113/jphysiol.1969.sp008719 PMC1350524

[12] Elek J, Prochazka A, Hulliger M, Vincent S. In‐series compliance of gastrocnemius muscle in cat step cycle: do spindles signal origin‐to‐insertion length? The Journal of Physiology. 1990;429(1):237–58. 2148952 10.1113/jphysiol.1990.sp018254 PMC1181697

[13] Lewis D, Proske U. The effect of muscle length and rate of fusimotor stimulation on the frequency of discharge in primary endings from muscle spindles in the cat. The Journal of Physiology. 1972;222(3):511 4260709 10.1113/jphysiol.1972.sp009812 PMC1331398

[14] Lennerstrand G, Thoden U. Dynamic Analysis of Muscle Spindle Endings in the Cat Using Length Changes of Different Length‐Time Relations. Acta Physiologica Scandinavica. 1968;73(1‐2):234–50. 4233732 10.1111/j.1748-1716.1968.tb04100.x

[15] Haftel VK, Bichler EK, Nichols TR, Pinter MJ, Cope TC. Movement reduces the dynamic response of muscle spindle afferents and motoneuron synaptic potentials in rat. Journal of Neurophysiology. 2004;91(5):2164–71. doi: 10.1152/jn.01147.2003 14695354 10.1152/jn.01147.2003

[16] Schäfer SS. The acceleration response of a primary muscle-spindle ending to ramp stretch of the extrafusal muscle. Cellular and Molecular Life Sciences. 1967;23(12):1026–7. 10.1007/BF02136428 4229611

[17] Schäfer SS, Kijewski S. The dependency of the acceleration response of primary muscle spindle endings on the mechanical properties of the muscle. Pflügers Archiv. 1974;350(2):101–22. 4277689 10.1007/BF00586231

[18] Boyd I. The response of fast and slow nuclear bag fibres and nuclear chain fibres in isolated cat muscle spindles to fusimotor stimulation, and the effect of intrafusal contraction on the sensory endings. Quarterly Journal of Experimental Physiology and Cognate Medical Sciences. 1976;61(3):203–53. 134389 10.1113/expphysiol.1976.sp002354

[19] Hasan Z. A model of spindle afferent response to muscle stretch. Journal of Neurophysiology. 1983;49(4):989 6222165 10.1152/jn.1983.49.4.989

[20] Lin C-CK, Crago PE. Structural model of the muscle spindle. Annals of Biomedical Engineering. 2002;30(1):68–83. 11874143 10.1114/1.1433488

[21] Mileusnic MP, Brown IE, Lan N, Loeb GE. Mathematical models of proprioceptors. I. Control and transduction in the muscle spindle. Journal of Neurophysiology. 2006;96(4):1772–88. doi: 10.1152/jn.00868.2005 16672301 10.1152/jn.00868.2005

[22] Nichols TR, Cope TC. Cross-bridge mechanisms underlying the history-dependent properties of muscle spindles and stretch reflexes. Can J Physiol Pharmacol. 2004;82(8–9):569–76. doi: 10.1139/y04-074 15523514 10.1139/y04-074

[23] Proske U, Stuart G. The initial burst of impulses in responses of toad muscle spindles during stretch. The Journal of Physiology. 1985;368(1):1–17. 2934546 10.1113/jphysiol.1985.sp015843 PMC1192582

[24] Proske U, Tsay A, Allen T. Muscle thixotropy as a tool in the study of proprioception. Exp Brain Res. 2014;232(11):3397–412. doi: 10.1007/s00221-014-4088-5 25200179 10.1007/s00221-014-4088-5

[25] Tsay A, Savage G, Allen TJ, Proske U. Limb position sense, proprioceptive drift and muscle thixotropy at the human elbow joint. Journal of Physiology. 2014;592(12):2679–94. doi: 10.1113/jphysiol.2013.269365 24665096 10.1113/jphysiol.2013.269365 PMC4080946

[26] Proske U, Gandevia SC. The kinaesthetic senses. The Journal of Physiology. 2009;587(17):4139–46. 19581378 10.1113/jphysiol.2009.175372 PMC2754351

[27] Campbell KS, Lakie M. A cross-bridge mechanism can explain the thixotropic short-range elastic component of relaxed frog skeletal muscle. Journal of Physiology. 1998;510 (Pt 3):941–62. 9660904 10.1111/j.1469-7793.1998.941bj.x PMC2231083

[28] Campbell KS, Moss RL. History-dependent mechanical properties of permeabilized rat soleus muscle fibers. Biophysical Journal. 2002;82(2):929–43. doi: 10.1016/S0006-3495(02)75454-4 11806934 10.1016/S0006-3495(02)75454-4 PMC1301901

[29] Campbell KS, Moss RL. A thixotropic effect in contracting rabbit psoas muscle: prior movement reduces the initial tension response to stretch. The Journal of Physiology. 2000;525(2):531–48. 10835052 10.1111/j.1469-7793.2000.00531.x PMC2269955

[30] Rack PM, Westbury D. The short range stiffness of active mammalian muscle and its effect on mechanical properties. The Journal of Physiology. 1974;240(2):331 4424163 10.1113/jphysiol.1974.sp010613 PMC1331019

[31] Morgan D. Separation of active and passive components of short-range stiffness of muscle. American Journal of Physiology-Cell Physiology. 1977;232(1):45–9. 10.1152/ajpcell.1977.232.1.C45 835695

[32] Proske U, Morgan D. Do cross-bridges contribute to the tension during stretch of passive muscle? Journal of Muscle Research & Cell Motility. 1999;20(5–6):433–42. 10555062 10.1023/a:1005573625675

[33] Hunt CC, Ottoson D. Impulse activity and receptor potential of primary and secondary endings of isolated mammalian muscle spindles. The Journal of Physiology. 1975;252(1):259–81. 127835 10.1113/jphysiol.1975.sp011143 PMC1348477

[34] Hunt C, Wilkinson R. An analysis of receptor potential and tension of isolated cat muscle spindles in response to sinusoidal stretch. The Journal of Physiology. 1980;302(1):241–62. 6447781 10.1113/jphysiol.1980.sp013240 PMC1282845

[35] Rack P, Westbury D. Elastic properties of the cat soleus tendon and their functional importance. The Journal of Physiology. 1984;347(1):479–95. 6231373 10.1113/jphysiol.1984.sp015077 PMC1199458

[36] Proske U, Morgan D. Tendon stiffness: methods of measurement and significance for the control of movement. A review. Journal of Biomechanics. 1987;20(1):75–82. 3558432 10.1016/0021-9290(87)90269-7

[37] Prochazka A, Gorassini M. Ensemble firing of muscle afferents recorded during normal locomotion in cats. The Journal of Physiology. 1998;507(1):293–304. 9490855 10.1111/j.1469-7793.1998.293bu.x PMC2230769

[38] Houk JC, Rymer WZ, Crago PE. Dependence of dynamic response of spindle receptors on muscle length and velocity. Journal of Neurophysiology. 1981;46(1):143–66. 6455505 10.1152/jn.1981.46.1.143

[39] Lee R, Tatton W. Motor responses to sudden limb displacements in primates with specific CNS lesions and in human patients with motor system disorders. Canadian Journal of Neurological Sciences/Journal Canadien des Sciences Neurologiques. 1975;2(03):285–93. 809129 10.1017/s0317167100020382

[40] Shemmell J, Krutky MA, Perreault EJ. Stretch sensitive reflexes as an adaptive mechanism for maintaining limb stability. Clinical Neurophysiology. 2010;121(10):1680–9. doi: 10.1016/j.clinph.2010.02.166 20434396 10.1016/j.clinph.2010.02.166 PMC2932821

[41] Matthews PB. Muscle spindles: their messages and their fusimotor supply. Comprehensive Physiology. 1981.

[42] Dimitriou M. Enhanced Muscle Afferent Signals during Motor Learning in Humans. Curr Biol. 2016;26(8):1062–8. doi: 10.1016/j.cub.2016.02.030 27040776 10.1016/j.cub.2016.02.030

[43] Dimitriou M. Human Muscle Spindle Sensitivity Reflects the Balance of Activity between Antagonistic Muscles. The Journal of Neuroscience. 2014;34(41):13644–55. doi: 10.1523/JNEUROSCI.2611-14.2014 25297092 10.1523/JNEUROSCI.2611-14.2014 PMC6608384

[44] Campbell KS. Dynamic coupling of regulated binding sites and cycling myosin heads in striated muscle. J Gen Physiol. 2014;143(3):387–99. doi: 10.1085/jgp.201311078 24516189 10.1085/jgp.201311078 PMC3933939

[45] Getz EB, Cooke R, Lehman SL. Phase transition in force during ramp stretches of skeletal muscle. Biophysical Journal. 1998;75(6):2971–83. doi: 10.1016/S0006-3495(98)77738-0 9826617 10.1016/S0006-3495(98)77738-0 PMC1299968

[46] Liu J-X, Eriksson P-O, Thornell L-E, Pedrosa-Domellöf F. Myosin heavy chain composition of muscle spindles in human biceps brachii. Journal of Histochemistry & Cytochemistry. 2002;50(2):171–83. 11799136 10.1177/002215540205000205

[47] Banks R, Barker D, Stacey M. Sensory innervation of cat hind-limb muscle spindles [proceedings]. The Journal of Physiology. 1979;293:40P–1P. 159358

[48] Jami L, Petit J. Dynamic and static responses of primary and secondary spindle endings of the cat peroneus tertius muscle. Journal of Physiology. 1979. 160926

[49] Bewick GS, Banks RW. Mechanotransduction in the muscle spindle. Pflügers Archiv-European Journal of Physiology. 2014:1–16. 10.1007/s00424-014-1536-9 PMC4281366 24888691

[50] Xiao R, Xu XS. Mechanosensitive channels: in touch with Piezo. Current Biology. 2010;20(21):R936–R8. doi: 10.1016/j.cub.2010.09.053 21056836 10.1016/j.cub.2010.09.053 PMC3018681

[51] Delmas P, Coste B. Mechano-gated ion channels in sensory systems. Cell. 2013;155(2):278–84. doi: 10.1016/j.cell.2013.09.026 24120130 10.1016/j.cell.2013.09.026

[52] Banks R, Hulliger M, Scheepstra K, Otten E. Pacemaker activity in a sensory ending with multiple encoding sites: the cat muscle spindle primary ending. The Journal of Physiology. 1997;498(1):177–99. 9023777 10.1113/jphysiol.1997.sp021850 PMC1159243

[53] Cui L, Perreault EJ, Maas H, Sandercock TG. Modeling short-range stiffness of feline lower hindlimb muscles. Journal of Biomechanics. 2008;41(9):1945–52. doi: 10.1016/j.jbiomech.2008.03.024 18499113 10.1016/j.jbiomech.2008.03.024 PMC2488157

[54] Kakuda N, Nagaoka M. Dynamic response of human muscle spindle afferents to stretch during voluntary contraction. The Journal of Physiology. 1998;513(2):621–8. 9807009 10.1111/j.1469-7793.1998.621bb.x PMC2231301

[55] Cordo PJ, Flores-Vieira C, Verschueren SM, Inglis JT, Gurfinkel V. Position sensitivity of human muscle spindles: single afferent and population representations. Journal of Neurophysiology. 2002;87(3):1186–95. 11877492 10.1152/jn.00393.2001

[56] Vallbo A. Human muscle spindle discharge during isometric voluntary contractions. Amplitude relations between spindle frequency and torque. Acta Physiologica Scandinavica. 1974;90(2):319–36. doi: 10.1111/j.1748-1716.1974.tb05594.x 4274638 10.1111/j.1748-1716.1974.tb05594.x

[57] Loram ID, Maganaris CN, Lakie M. Paradoxical muscle movement in human standing. The Journal of Physiology. 2004;556(3):683–9. 15047776 10.1113/jphysiol.2004.062398 PMC1664994

[58] De Groote F, Allen JL, Ting LH. Contribution of muscle short-range stiffness to initial changes in joint kinetics and kinematics during perturbations to standing balance: A simulation study. Journal of Biomechanics. 2017;55:71–7. doi: 10.1016/j.jbiomech.2017.02.008 28259465 10.1016/j.jbiomech.2017.02.008 PMC5436583

[59] Insperger T, Milton J, Stépán G. Acceleration feedback improves balancing against reflex delay. Journal of the Royal Society Interface. 2013;10(79):20120763. 10.1098/rsif.2012.0763 PMC3565692 23173196

[60] Boyd I, Gladden MH, McWilliam P, Ward J. Control of dynamic and static nuclear bag fibres and nuclear chain fibres by gamma and beta axons in isolated cat muscle spindles. The Journal of Physiology. 1977;265(1):133–62. 139469 10.1113/jphysiol.1977.sp011709 PMC1307812

[61] Welch TD, Ting LH. Mechanisms of motor adaptation in reactive balance control. PLoS One. 2014;9(5):e96440 doi: 10.1371/journal.pone.0096440 24810991 10.1371/journal.pone.0096440 PMC4014487

[62] Proske U, Gandevia SC. The proprioceptive senses: their roles in signaling body shape, body position and movement, and muscle force. Physiol Rev. 2012;92(4):1651–97. doi: 10.1152/physrev.00048.2011 23073629 10.1152/physrev.00048.2011

[63] Jami L, Petit J, Proske U, Zytnicki D. Responses of tendon organs to unfused contractions of single motor units. Journal of Neurophysiology 1985;53(1):32–42. 3973662 10.1152/jn.1985.53.1.32

[64] Jami L. Golgi tendon organs in mammalian skeletal muscle: functional properties and central actions. Physiological Reviews. 1992;72(3):623–66. 1626033 10.1152/physrev.1992.72.3.623

[65] Day J, Bent LR, Birznieks I, Macefield VG, Cresswell AG. Muscle spindles in human tibialis anterior encode muscle fascicle length changes. Journal of Neurophysiology. 2017;117(4):1489–98. doi: 10.1152/jn.00374.2016 28077660 10.1152/jn.00374.2016 PMC5376612

[66] Horslen BC, Murnaghan CD, Inglis JT, Chua R, Carpenter MG. Effects of postural threat on spinal stretch reflexes: evidence for increased muscle spindle sensitivity? Journal of Neurophysiology. 2013;110(4):899–906. doi: 10.1152/jn.00065.2013 23719208 10.1152/jn.00065.2013

[67] Hwang EJ, Shadmehr R. Internal models of limb dynamics and the encoding of limb state. Journal of Neural Engineering. 2005;2(3):S266 doi: 10.1088/1741-2560/2/3/S09 16135889 10.1088/1741-2560/2/3/S09 PMC1479856

[68] Blum KP, D'Incamps BL, Zytnicki D, Ting LH. Data from: Force encoding in muscle spindles during stretch of passive muscle. Dryad Digital Repository; 2017 doi: 10.5061/dryad.pd40m 10.1371/journal.pcbi.1005767 PMC5634630 28945740

[69] Burnham KP, Anderson D. Model selection and multi-model inference: A Pratical information-theoretic approch. Second Edition Springer 2003.

